# Indications and Practical Application of Local Anesthesia in Dentistry*Read before the Chicago Dental Society and reprinted from the *Dental Review* of February, 1918.

**Published:** 1918-02

**Authors:** P. G. Puterbaugh

**Affiliations:** Chicago, Ill.


### INDICATIONS AND PRACTICAL APPLICATION OF LOCAL ANESTHESIA IN DENTISTRY *

BY P. G. PUTERBAUGH, M.D., D.D.S., CHICAGO, ILL.

The subject of the alleviation of pain is one of the oldest in the history of medicine, dating from the misty ages when certain criminals who were sentenced to the lash drank decoctions of stupefying drugs in order to render the perception of its sting less acute, down through the centuries until only within the last seventy-five years has anesthesia become a reality. It is true that refrigeration by means of ice packs, ether and ethyl chlorid sprays, pressure upon nerve trunks, etc., has been employed for many years, but it was not until 1884, with the discovery of the anesthetic properties of cocain, that true local anesthesia, as we now understand the term, was induced. In the following year, 1885, Halstedt successfully anesthetized the mandible, using conductive anesthesia with cocain. Local and spinal anesthesia were practiced widely for a time, and, while

* Read before the Chicago Dental Society and reprinted from the *Dental Review* of February, 1918.

the anesthesia left little to be desired, the occasional toxic symptoms attendant upon the cocain injections caused many operators to abandon the practice of local for nitrous oxid or ether anesthesia by inhalation.

The peculiar situation of the mouth with its intimate connection with the respiratory passages, however, renders continuous anesthesia a difficult matter by any inhalation method. Because of the unsatisfactory induction too frequently met with in attempts to administer general anesthesia for dental operations in the routine of a busy practice, the average dentist, in the past, has evaded the use of general anesthesia in all except those presenting definite and positive indications for its administration, the choice always being in favor of local anesthesia if it could be satisfactorily induced.

Because of the initial pain accompanying the insertion of the needle, because of toxic symptoms observed, because of after pain and soreness, and because of occasional sloughing of tissue and even abscess formation occurring at the site of injection, many otherwise conscientious operators have shunned the hypodermic syringe and the local anesthetic solution, thereby inflicting untold agonies that might have been avoided had they mastered the technic of our modern methods of induction. Every dentist can do better and more thorough work if he can operate painlessly, and, incidentally, patients may use up their nervous energy in much more useful pursuits than that of enduring unnecessary pain incident to the excavation of sensitive cavities, the extirpation of vital pulps or the extraction of teeth.

Numerous cocain substitutes have been offered by various chemists from time to time, but after short trials all presented more or less serious objections until the discovery of novocain by Einhorn in 1905. After twelve years of extensive use in all branches of surgery, novocain today fills the requirements of the ideal local anesthetic.

Novocain is nontoxic in the required dosage, nonirritating to tissue, stable enough to withstand sterilization by boiling, and is capable of uniting temporarily with nervous tissue in a manner to produce an anesthesia that is absolutely satisfactory to both patient and operator.

In overcoming the disagreeable sequellæ that have too often followed the administration of local anesthetics in the past, we found that certain modifications were necessary: First, in the local anesthetic solution, and second, in the carrying of the solution to the tissues. The human body being composed of a high percentage of water, makes it logical to employ that "universal solvent" as the vehicle for carrying the anesthetic agent to the tissues that we wish to influence.

Chemically, sterile distilled water presents no properties injurious to the tissues beyond that of disturbing the osmotic balance between the tissue cells and their surrounding fluids; a feature that may be readily overcome. If definitely uniform results are to be expected, the use of freshly distilled water is essential, for the reason that deterioration takes place in distilled water upon standing. The effect of this is observable in the collection of sediment in the bottom of the container in the form of a flocculent precipitate. This decomposition may be aided by the action of light, bacterial organisms, or chemical salts entering into the composition of the glass container. The anesthetic solutions compounded from distilled water that has become stale are more irritating than those prepared from freshly distilled water has been observed by many operators. Therefore, let us start right in the preparation of our solutions by purchasing freshly distilled water, or what is preferable, employing one of the small, inexpensive stills obtainable from any dental or surgical supply house and distilling the amount required as it is needed.

Physiologists long ago observed that the bathing of tissues in unmodified distilled water caused a swelling of the individual cells, due to the disturbed osmotic balance; the tissues containing normally a definite percentage of crystalloid material in the form of salts, chlorids of sodium, potassium and calcium. The presence of these salts caused the hypotonic distilled water to be imbibed by the tissue cells, resulting in mechanical injury to the delicate stroma and cytoplasm itself. This disturbance within the cells composing any tissue injected with a hypotonic anesthetic solution is in itself sufficient to account for much of the smarting pain, swelling and after soreness so frequently observed following the administration of certain widely advertised proprietary solutions. These disagreeable symptoms may be avoided by the addition of the proper amount of salts to the water, rendering the osmotic balance equal to that of the human tissues. The so-called Ringer solution fills this need satisfactorily, having the formula of—

| | |
|---|---|
| Sodium chlorid ........... | 0.6 per cent. |
| Calcium chlorid ........... | 0.4 per cent. |
| Potassium chlorid .......... | 0.2 per cent. |

For convenience in compounding, the ingredients in proper proportions have been incorporated in tablet form by various pharmaceutical houses, rendering the making of the Ringer solution a very simple matter. All that is necessary is to add a tablet to a measured quantity of distilled water. This solution may be injected hypodermically without pain, discoloration, edema or after soreness, save that resulting from the introduction of the needle, because it does not disturb the osmotic balance of the cells with which it comes in contact and is positively nonirritating when freshly prepared.

Novocain, a white crystalline powder, synthetically prepared, is freely soluble in water in all dilutions up to equal parts. It is nonirritating, even when the pure crystals are dusted upon abraded surfaces; it is vaso-dilator to a very slight degree, this effect being overcome by the addition of suprarenin, and it is nonhabit forming. Novocain possesses the property of combining temporarily with nervous tissue in such a manner that it causes a suspension of its power of receiving or of transmitting impulses during that period of combination, and of leaving no perceptible tissue change after its effect has been dissipated. It is rated as being seven times less toxic than cocain in the average individual, while idiosyncrasies to novocain are extremely rare. Statistics of the repeated injections of amounts of three to six ounces of a one per cent. solution and from one-half to one ounce of a two per cent. solution without untoward effects place the comparatively small amounts required for dental cases entirely within the limits of absolute safety.

In my work I find that the one per cent. solution for infiltrative anesthesia in minor surgery, such as simple extractions, pulp extirpations, etc., and for conductive anesthesia in the upper jaw, where the nerve trunks are of small diameter, leaves nothing to be desired; but for conductive anesthesia of the heavier mandibular nerve a two per cent. solution should be used, as it will reach the central fibers of the trunk in sufficient amount to give a complete anesthesia of the deeper structures, including the pulps of the teeth. If the one per cent. solution be used here an anesthesia of the lip and soft tissues supplied by the outer fibers would ensue, but the central fibers being unaffected by the weak solution would result in a failure to anesthetize the dental pulps of the posterior teeth.

Following the isolation of the blood-pressure-raising principle of the suprarenal gland with a study of its physiologic action and the ischemia produced by its local application due to its vaso-constrictor properties, it was suggested that the combination of adrenal solutions with local anesthetic agents would tend to localize and thus intensify and prolong their effect by preventing the rapid dissipation into the systemic circulation. Clinical evidence has conclusively proven this to be true, while it also was found to possess the additional advantage of preventing toxic symptoms by retarding its absorption into the systemic circulation. Thus it has been demonstrated that cocain may be employed in one-half of one per cent. solution when combined with adrenalin with results equal to a two per cent. solution, in so far as the anesthesia is concerned, with much less danger from cocain poisoning.

The organic product obtained from suprarenal glands offers a somewhat unstable preparation; but in recent years a synthetic preparation of identical chemical formula has been marketed that has better keeping qualities than the organic product and being obtainable in tablet form is convenient for use.

Suprarenin, when introduced into the circulation, causes an increase in the force and frequency of the heart action, accompanied by a marked rise in blood pressure, due to a direct action upon the musculature rather than to stimulation of the central nervous system. Locally, it constricts the smaller blood vessels by direct stimulation of the circular muscle fibers in their walls. Upon injection hypodermically, this vaso-constrictor action is evidenced by an ischemic area surrounding the site of injection for a distance of one to two centimeters within from five to ten seconds and lasts from thirty to sixty minutes. After a time, the effect wearing away, the condition of vaso-dilatation ensues for several hours; probably due to exhaustion of the muscular tunics from over-stimulation. Suprarenin, if administered in an overdosage, produces the quite characteristic and distressing signs and symptoms of precordial pain, air hunger, dizziness, a very full and often intermittent pulse, headache, etc. These symptoms come on suddenly, but are very transient, lasting only from three to ten minutes, and are those of an overstimulation and increased blood pressure, but are not followed by periods of depression, such as is observed following cocain toxemias. In the two cases that I have observed no treatment was given, the symptoms soon subsiding and the operations proceeding without further difficulty. These symptoms may, I believe, be averted by following the slow injection method. Just as the point of the needle enters the tissues, expel two or three drops of solution and then wait a few seconds to allow the vaso-constrictor action to take place before continuing. The barrier formed by the ischemic ring resulting from the primary injection walls off and prevents systemic intoxication, and incidentally increases the profundity of the anesthesia by retaining the solution in the injected area.

One other requisite of local anesthetic solutions is that they be rendered sterile. Many antiseptics have been employed for this purpose, the majority of them being used with the idea of acting as preservatives of the solution. Unfortunately, the ideal anesthetic solution can not be preserved successfully for more than a few days, and all of the preservative agents are open to criticism, inasmuch as the anesthetic solutions are less irritating if they are omitted. A freshly prepared solution may be boiled for a short time, thus rendering it sterile, without impairing its action in the least, and is to be recommended over the chemical preservatives that have been suggested from time to time. The practice of adding cardiac, cerebral or respiratory stimulants, followed for so many years, is looked upon today as unwarranted and should be discouraged.

To briefly review, our anesthetic solution is now composed as follows: Freshly distilled water as a vehicle, one to two per cent. novocain as the anesthetic agent, sufficient chlorids of sodium, calcium and potassium in the form of a Ringer tablet to render the solution isotonic with the body fluids, sufficient suprarenin to localize and retain the anesthetic mixture in the locality in which the injection is made, and the solution thus compounded, sterilized by boiling.

For simplicity, all of the ingredients have been included in one tablet, but these show indisputable evidence of deterioration after being kept for a few weeks so they are not to be depended upon. However, time has shown that novocain and synthetic suprarenin may be included in the same tablet without deterioration and Ringer solution tablets may likewise be kept indefinitely. By the use of these two tablets and freshly distilled water we are enabled to prepare a solution in a few minutes that will cut short the patient's discomfort many hours when compared to the stock solutions too often employed, and the absence of after soreness and pain fully compensate for the extra effort expended.

Body cells are just as much subject to "catching cold" or of having their resistance lowered by cold as our respiratory mucosa, and the chilling of the site of injection by carrying a cold solution into the tissues is just as productive of damage as is extreme cold from any cause. This injury with consequent lowering of resistance and retarding of tissue repair may be readily avoided by the injection of warm solutions only. If the anesthetic solution be drawn into the hypodermic syringe immediately after boiling, and the injection proceeded with, it will be found to be just about the body temperature by the time it reaches the tissues. This is an important item that has been overlooked too often in our local anesthetic operations.

Then, too, we must reckon on the amount of force required for injection, ever bearing in mind the excessive force that would traumatize and injure tissue. I have seen a number of instances in which sloughing of areas as large as a five cent piece occurred. These are seen most frequently in the hard palate and in the lower first molar region, and were traceable directly to the hydraulic pressure exerted by the forcing of an anesthetic solution therein with a heavy syringe. We must bear in mind that fixed tissue cells are bathed in lymph, and that serious damage is sure to result unless time be allowed during the injection for the lymph to escape ahead of the anesthetic solution as it is forced in; and in order to avoid mechanical injury, only very light pressure should be exerted upon the piston during the injection, and if 60-70 seconds are consumed in emptying the syringe, the patient is thereby the gainer.

Oral mucosa, like the skin, constantly harbors infectious bacterial organisms in its superficial layers. These are held in abeyance under normal conditions by the phagocytic properties of the leucocytes, but if they be carried mechanically into the deeper structures, inflammatory processes would likely ensue. This carrying of infectious material along the course of the needle has undoubtedly been the cause of considerable after soreness, pain and delayed healing following the administration of local anesthetics and should be considered as one of the avoidable accidents.

Tincture of iodin U. S. P. 7 per cent., applied to the mucosa by means of a tightly rolled pellet of cotton and massaged well into the tissues at the point selected for the insertion of the needle, will not only disinfect the site, but will render the pain of the initial puncture with the needle almost imperceptible.

With a little practice almost any operator may so train himself to manipulate the hypodermic syringe that the pain of the insertion may be still further minimized by holding the syringe during its insertion in such a manner that no change in the grasp need be made in order to expel its contents. The best method will be found to be the balancing of the syringe with the barrel resting between the second and third fingers of the right hand, placing the first and fourth fingers above the second and third respectively with the cross bars resting between, and the thumb on the piston. Holding the syringe in this manner permits of the injection of two or three drops of anesthetic solution just as the needle enters the mucous membrane and allows the injection to proceed while it is advanced through the tissues, thus anesthetizing ahead of the needle as it is carried in.

The site selected for the primary insertion of the needle should, when conditions permit, be at the point of reflection of the buccal or labial mucosa from the alveolar process. Mucous membrane at this point, because of its protection from irritation, has a much poorer sensory nerve supply than the mucous membrane of the gingivae, and by taking advantage of this we may make the needle insertion at that point with a minimum amount of pain. The submucosa here is loosely constructed, with large intercellular lymph spaces, allowing the injection of anesthetic solution with very little pressure, there being an easy interchange of anesthetic for the lymph surrounding the fixed tissue cells, giving true anesthesia without such disastrous complications as tearing or traumatizing of tissue.

One of the most frequent causes of imperfect anesthesia that is met with today is that the operator, after making a satisfactory injection, will proceed with the operation immediately without waiting for the anesthetic agent to act upon the nerve fibers. All of the local anesthetic agents in common use require from five to eight minutes contact with nerve endings or nerve fibers in order to be absorbed and to reach the maximum anesthesia. Therefore, when we allow for diffusion throughout the injected area and sufficient time for the drug to be absorbed and reach the maximum anesthesia possible, ten minutes is not too long to wait for a true anesthesia of any of the smaller nerve fibers, and from fifteen to twenty minutes are needed for larger trunks such as the mandibular and posterior dental nerves.

If anesthesia be obtained following either infiltrative or conductive injections, it may be safely assumed that it is a pressure anesthesia from tissue injury produced by using too much force during the injection rather than from any action of the anesthetic agent employed, and would be obtained just as certainly if plain water had been employed instead. This condition is to be avoided because a condition of lowered resistance is produced from the injury, accompanied by after soreness and edema and is marked by delayed healing.

Local anesthesia is classified under two distinct divisions, viz.: infiltrative, the application of a local anesthetic agent to the distribution of sensory nerves, thus paralyzing their endings and their power of receiving impressions, while conductive anesthesia consists of the injection of a local anesthetic solution into the region surrounding a sensory nerve trunk, thus paralyzing its power of transmitting afferent impulses sent up from its area of distribution.

From many procedures the infiltrative type of anesthesia is perfectly satisfactory, for example, the removal of normal or hypertrophied gum from overerupting teeth or from proximal cavities that it has filled in, or of hypertrophied pulps the injection may be made directly into the tissue to be excised so that the point where the puncture is made is excised with the tissue, thereby avoiding any after soreness from that cause, as would probably occur to a slight degree if the injection be made around the area. For the removal of superficially seated roots that have little bony process supporting them, the infiltration may be made by injecting to the buccal and lingual surfaces, allowing sufficient time for the maximum anesthesia to ensue. For cavity preparation in the lower incisors or the ten upper anterior teeth, the infiltration of 1 cc of solution over the apices of the roots will usually obtund sensation. For the extraction of the lower incisor teeth infiltrative anesthesia suffices and is preferable to conductive, since the latter anesthesia often affects the entire side of the face for two or three hours. For lancing abscesses where pus has localized and palpation elicits fluctuation, the infiltration of the mucous membrane only, in the line of incision, using care not to allow the needle to enter the abscess cavity, will render the operation painless. The maximum anesthesia here requires waiting for from three to five minutes after injecting. For the extraction of deciduous teeth, the roots of which are not deeply seated, a small amount of one per cent. novocain solution infiltrated about the teeth of controllable children will give perfect anesthesia unless abscess conditions be present.

Almost everyone who has seriously entered the practice of conductive anesthesia has found that with increasing experience in its use he employs it more and more to the exclusion of the commonly practiced infiltrative type. This is accounted for in the comparative painlessness of the needle insertion and the injection, the small amount of solution required, the absence of after pain and soreness and the absolute anesthesia obtained. Patients upon whom it has been used for cavity preparation or surgical procedures are pleased with the results and request its use on subsequent occasions, and that encourages one to use it with increasing frequency as time goes on. It is not recommended over, nor will it ever supplant nitrous oxid for the highly nervous, uncontrollable neurasthenic who dreads operations; for the physically exhausted individual who has been in pain for a number of days; for the child with whom it is difficult to reason, nor for extractions in acute abscess conditions. But for the preparation of the sensitive cavity that does not respond to the ordinary methods of desensitization; for the removal of vital pulps; for root amputations; for extractions; for prolonged and complicated operations such as the removal of unerupted and impacted teeth; for maxillary sinus operations; for the reduction of fractures of the mandible and for the removal of tumors and cysts about the jaws it gives complete anesthesia. The patient can be placed in any position in the dental chair that is most convenient to the operator; there is no aspiration or swallowing of blood or mucus and no nausea, vomiting or prostration after the operation is over. In fact, the anesthesia will usually last until the patient reaches home, which in itself is highly desirable.

In conclusion, allow me to say that we are working in a new era in local anesthesia; that he who practices it most successfully will be the man who observes to the last detail the fundamental principles that I have attempted to briefly outline.

